# A Novel Mitosomal β-Barrel Outer Membrane Protein in *Entamoeba*

**DOI:** 10.1038/srep08545

**Published:** 2015-02-25

**Authors:** Herbert J. Santos, Kenichiro Imai, Takashi Makiuchi, Kentaro Tomii, Paul Horton, Akira Nozawa, Mohamed Ibrahim, Yuzuru Tozawa, Tomoyoshi Nozaki

**Affiliations:** 1 Department of Parasitology, National Institute of Infectious Diseases, 1-23-1 Toyama, Shinjuku-ku, Tokyo 162-8640, Japan; 2 Graduate School of Life and Environmental Sciences, University of Tsukuba, 1-1-1 Tennodai, Tsukuba, Ibaraki 305-8572, Japan; 3 Institute of Biology, College of Science, University of the Philippines Diliman, Quezon City, 1101 Philippines; 4 Computational Biology Research Center (CBRC), National Institute of Advanced Industrial Science and Technology (AIST), 2-4-7 Aomi, Koto-ku, Tokyo 135-0064, Japan; 5 Department of Infectious Diseases, Tokai University School of Medicine, Isehara, Kanagawa 259-1193, Japan; 6 Proteo-Science Center, Ehime University, 3 Bunkyo-cho, Matsuyama, Ehime 790-8577, Japan; 7 Botany Department, Faculty of Science, Ain Shams University, Khalifa El-Maamon St, Abbasiya Sq., Cairo, 11566, Egypt; 8 Graduate School of Science and Engineering, Saitama University, 255 Shimo-Okubo, Sakura-ku, Saitama, Saitama 338-8570, Japan

## Abstract

*Entamoeba* possesses a highly divergent mitochondrion-related organelle known as the mitosome. Here, we report the discovery of a novel protein in *Entamoeba*, which we name Mitosomal β-barrel Outer Membrane Protein of 30 kDa (MBOMP30). Initially identified through *in silico* analysis, we experimentally confirmed that MBOMP30 is indeed a β-barrel protein. Circular dichroism analysis showed MBOMP30 has a predominant β-sheet structure. Localization to *Entamoeba histolytica* mitosomes was observed through Percoll-gradient fractionation and immunofluorescence assay. Mitosomal membrane integration was demonstrated by carbonate fractionation, proteinase K digestion, and immunoelectron microscopy. Interestingly, the deletion of the putative β-signal, a sequence believed to guide β-barrel outer membrane protein (BOMP) assembly, did not affect membrane integration, but abolished the formation of a ~240 kDa complex. MBOMP30 represents only the seventh subclass of eukaryotic BOMPs discovered to date and lacks detectable homologs outside *Entamoeba*, suggesting that it may be unique to *Entamoeba* mitosomes.

Mitochondria can possess highly divergent and often degenerate morphology, function, and components in eukaryotes that are adapted to anoxic or hypoxic environments. In cases in which morphology is drastically changed and some hallmark mitochondrial processes such as oxidative phosphorylation, the TCA cycle, and β-oxidation are lost, these organelles are called Mitochondrion-Related Organelles (MROs), or specifically, hydrogenosome and mitosome. Mitosomes are particularly degenerate organelles, lacking cristae structure, and even the ability to synthesize ATP. It is believed that mitosomes, as well as hydrogenosomes, have occurred multiple times during eukaryotic evolution because organisms that possess mitosomes do not cluster together in eukaryote phylogenies, and the size, function, and content of mitosomes differ between organisms^1^,^2^,^3^. Like Gram-negative bacteria and chloroplasts in primary land plants, mitochondria and MROs possess a double membrane. Transport of proteins and metabolites across the outer membrane is mediated by pore-forming β-barrel Outer Membrane Proteins. (Hereafter we use “BOMP” to denote any β-barrel outer membrane protein and “MBOMP” to denote BOMPs from mitochondria and MROs).

In mitochondria, six subclasses of MBOMPs have been previously identified: Tom40, Sam50, VDAC, Mdm10, ATOM and Tac40. Tom40 is the core pore component of the Translocase of the Outer Membrane (TOM) complex required for the import of mitochondrial precursor proteins into mitochondria^4^,^5^. Sam50 is the central component of the Sorting and Assembly Machinery (SAM) complex and promotes the integration of MBOMPs^6^,^7^,^8^ to the outer membrane. Both Tom40 and Sam50 are essential for yeast viability^4^,^5^,^8^. VDAC (Voltage-Dependent Anion Channel) primarily serves as a non-specific diffusion pore for small molecules entering or leaving the mitochondria^9^. Mdm10 (Mitochondrial Dynamics and Morphology 10) has only been clearly identified in fungi, and is involved in mitochondrial morphogenesis and dynamics^10^, as well as in the biogenesis of mitochondrial BOMPs as it was reported to be a part of the SAM complex^11^. It is also a member of the ER-mitochondria tethering complex known as the ER-Mitochondria Encounter Structure, ERMES^12^. Trypanosomatids lack Tom40, and instead have a unique translocase called ATOM^13^ (Archaic Translocase of the Outer Mitochondrial membrane). Recently another trypanosome-specific MBOMP Tac40, a member of the Tripartite Attachment Complex, was identified. Tac40 also belongs to the mitochondrial porin family, and is essential to mitochondrial DNA inheritance, as it physically links the mitochondrial genome to cytoskeletal components of both the mitochondrion and flagellum of *Trypanosoma brucei*^14^. Among all MBOMPs, only Sam50 and ATOM have recognizable homologs outside of the Eukaryota. Sam50 has a bacterial homolog, Omp85/BamA, a 16-stranded bacterial BOMP^15^, which also integrates and assembles BOMPs^16^; while the homolog of ATOM belongs to the Omp85 subgroup called YtfM^13^, which is a BOMP required for normal growth in *Escherichia coli*^17^.

*Entamoeba histolytica* is an anaerobic unicellular parasite that causes dysentery and extra-intestinal abscesses that are responsible for an estimated 100,000 deaths annually. This organism possesses highly divergent mitosomes, as predicted by a recent proteomic study^18^. It appears that the mitosome proteome in *E. histolytica* is far less complex than mitochondria (e.g., yeast mitochondria are believed to harbor around 1000 proteins^19^,^20^), and remarkably different from other MROs. Indeed, even Fe-S cluster biogenesis, which is the only known common function of mitochondria and MROs, is uncertain in *E. histolytica* mitosomes^21^,^22^. It was reported that the iron-sulfur cluster assembly genes *iscS* and *iscU* of *E.* *histolytica* were acquired by horizontal gene transfer^22^,^23^ and unlike other organisms, *E. histolytica*^21^,^22^ and the distantly related *Mastigamoeba balamuthi*^24^ use a NIF (nitrogen fixation)-like system for Fe-S cluster biogenesis; which is primarily, if not exclusively, cytosolic and appears to have been obtained by horizontal transfer from ε-proteobacteria. Thus far, the only established role of the *Entamoeba* mitosome is sulfate activation^18^,^25^.

Among the many “missing links” in understanding mitosomal biology is the conspicuous absence of detectable homologs to VDAC, an MBOMP which is the usual channel for metabolites in the mitochondrial outer membrane. Intrigued by this and encouraged by the fact that our previously developed method^26^,^27^ for predicting MBOMPs from amino acid sequence had already been able to predict a candidate novel plastid BOMP TGD4 (At3g06960), subsequently confirmed to localize to the plastid outer membrane^28^; we endeavored to combine our informatics and experimental techniques to search for novel *Entamoeba* BOMPs. These efforts led us to discover MBOMP30, a novel lineage-specific BOMP which localizes to the outer membrane of the *E. histolytica* mitosome.

## Results

### *In silico* screening of novel MBOMP candidates in *E. histolytica*

**Figure 1:**
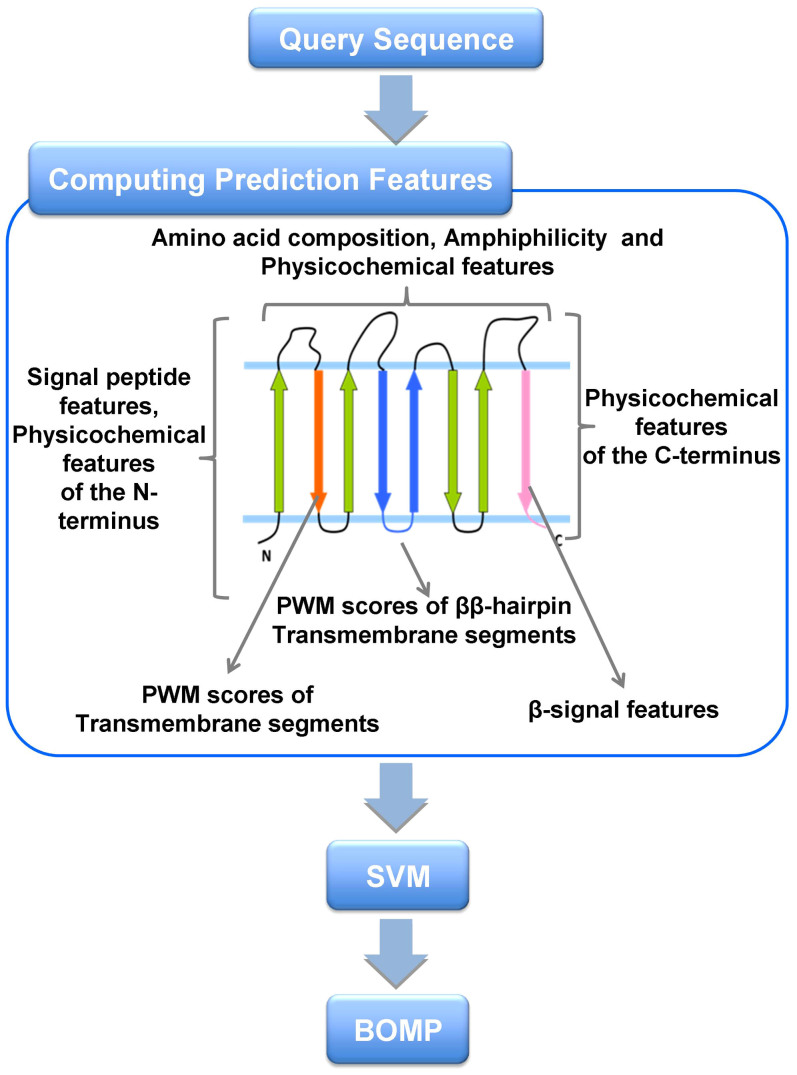
The architecture of our MBOMP prediction system. Sequence features used by our MBOMP predictor are listed in relation to a schematic depiction of MBOMP structure.

To identify novel MBOMP candidates in *E. histolytica*, we customized our MBOMP predictor^27^ for MRO's, and also refined the screening method in general (see Materials and methods) (Fig. 1, Supplementary Table S1). We screened 8,306 proteins in the *E. histolytica* genome database using an updated version of our MBOMP prediction pipeline, yielding six MBOMP candidates: EHI_178630, EHI_007460, EHI_163510, EHI_050690, EHI_068370, and EHI_104420 (Table 1). EHI_104420 is a homolog of Tom40, which has been previously characterized^29^. Four of the other five proteins are annotated as non-mitochondrial proteins and have sequence similarity to known functional domains. Thus, in this study, we chose to focus on the remaining protein EHI_178630 (Uniprot accession C4LUS8), which attained the second highest predictor score, second only to the known MBOMP Tom40, and we hereafter denote as *E. histolytica* MBOMP30 (Mitosomal β-barrel Outer Membrane Protein of 30kDa).

### MBOMP30 has no detectable homologs outside of *Entamoeba*

To examine the phylogenetic distribution of MBOMP30 among the three domains of life, we used the highly sensitive iterative profile HMM comparison search methods JackHMMR and HHblits^30^,^31^ to search for homologs of *E. histolytica* MBOMP30 in other organisms. We found no significant hits (E-value < 1e-5) in either bacteria or archaea. Among eukaryotes, we found homologs only in genus *Entamoeba*, namely, *E. nuttalli*, *E. dispar* and *E. invadens*. Notably, we found no hits in the relatively well-characterized free-living aerobic amoebozoan *Dictyostelium discoideum*. Both highly related to *E. histolytica, E. nuttalli*, a pathogenic parasite of non-human primates, and *E. dispar*, a non-pathogenic species, possess very similar sequences ENU1_140620 and EDI_035580, alignable to MBOMP30 at 97.2% and 86.5% identity (although EDI_035580 is truncated), respectively. On the other hand, the more distant reptilian parasite *E. invadens* yielded a more diverged, but still highly significant hit EIN_041060 (with an identical sequence assigned as EIN_066350 in NCBI), having a 32.8% identity (E-value 1.8e-27) (Supplementary Table S2).

### MBOMP30 is predicted to contain transmembrane β-strands

**Figure 2:**
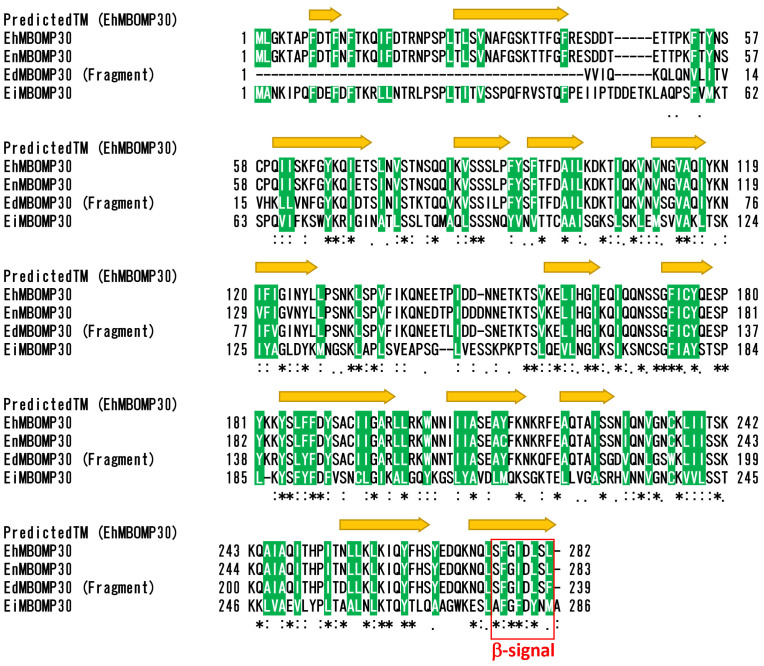
Prediction of transmembrane β-strands of EhMBOMP30 and multiple alignment with its homologs. Multiple alignment of EhMBOMP30, EnMBOMP30, EdMBOMP30, and EiMBOMP30, built with Clustal omega^59^ is shown in CLUSTAL format (Eh-*Entamoeba histolytica*, En-*Entamoeba nuttalli*, Ed-*Entamoeba dispar*, Ei-*Entamoeba invadens*). Predicted transmembrane β-strands of EhMBOMP30 are indicated with yellow arrows. The arrows cover all positions predicted as part of a β-strand by either of two BOMP topology predictors: BOCTOPUS and TMBETAPRED-RBF^32^,^33^ [See Fig. S1]. Conserved hydrophobic residues are highlighted in green. A red dashed box indicates the region corresponding to the β-signal motif in the last predicted transmembrane β-strand.

Our MBOMP predictor gives a high probability score for all four *Entamoeba* MBOMP30 homologs. To assure that this result was not a quirk of our predictor, we analyzed the *E. histolytica* MBOMP30 sequences using two tools designed for topology prediction of bacterial BOMPs, which may be expected to share some structural properties with MBOMPs. The topology prediction tools BOCTOPUS and TMBETAPRED-RBF^32^,^33^ predicted the *Entamoeba* MBOMP30 to contain multiple transmembrane β-strand regions (Fig. 2, Supplementary Fig. S1a), consistent with the premise that MBOMP30 is a mitosomal BOMP. The outside surface of MBOMPs faces a lipid environment and is expected to display a relatively high hydrophobicity. The MBOMP30 sequences indeed share this property, and their predicted β-strand regions and hydrophobic stretches align well (Supplementary Fig. S1b).

### Far-UV spectroscopy and circular dichroism (CD) suggests high β-strand content of *E. histolytica* MBOMP30

**Figure 3:**
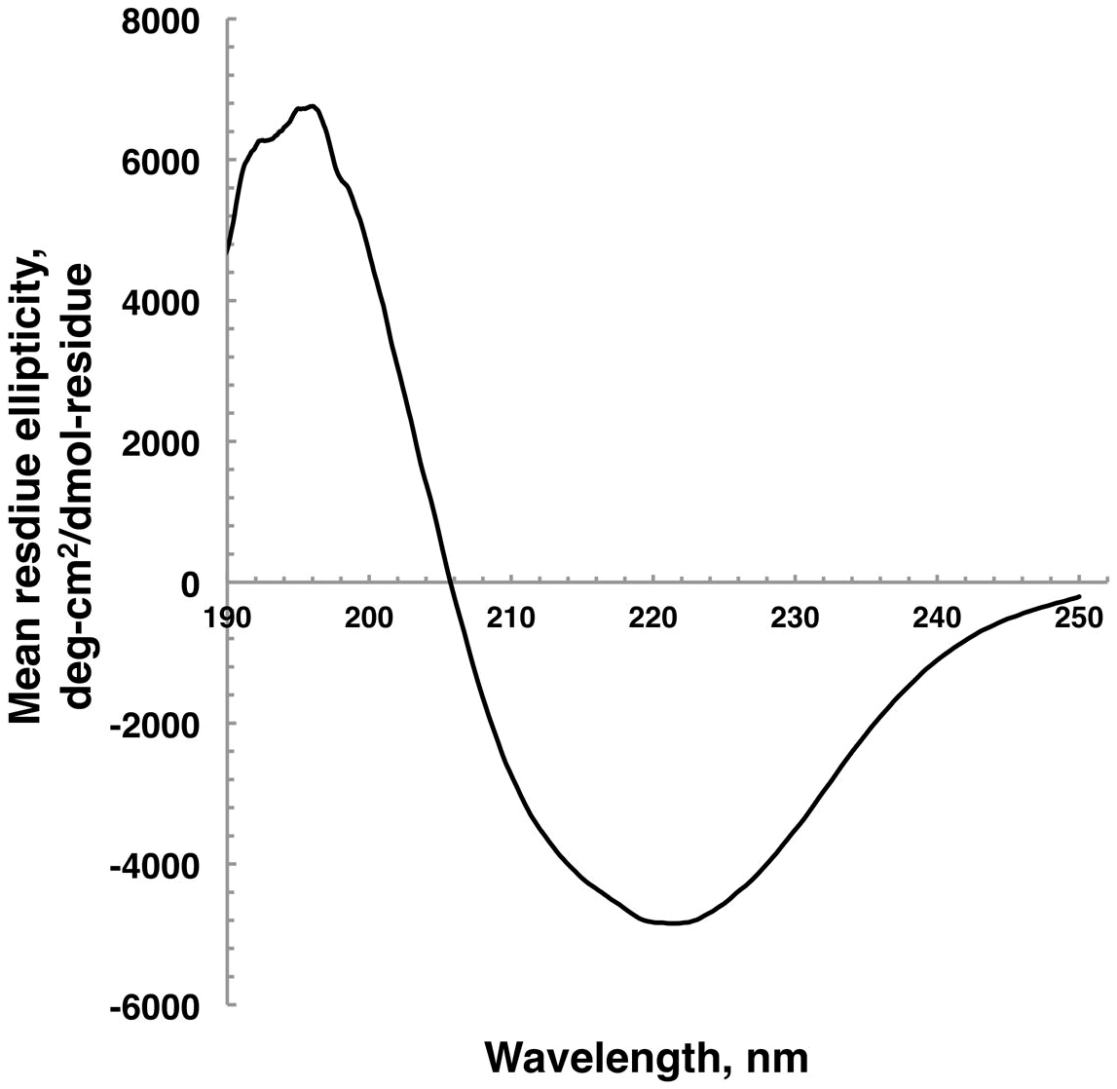
Circular dichroism spectroscopy. The far-ultraviolet CD spectra of MBOMP30 in DPhPc liposomes, expressed in a cell-free system, were taken from the accumulation of 9 consecutive scans.

We synthesized *E. histolytica* MBOMP30 by wheat germ cell-free expression system in the presence of 1,2-diphytaynyl-sn-glycero-3-phosphocholine (DPhPC) liposomes (Supplementary Fig. S2), and estimated the secondary structure composition of the protein by CD spectroscopy. The *E. histolytica* MBOMP30 far-UV spectra indicated a pattern representative of β-strand-rich proteins, having minimum and maximum ellipticities near 220 and 195 nm, respectively (Table 2, Fig. 3). This was similar to that of a β-barrel control, green fluorescent protein^34^. In addition, using the CONTIN algorithm, the deconvoluted CD spectra predicted that *E. histolytica* MBOMP30 has a high β-strand-content estimated at 30.5 ± 2.9%, while having only 14.1 ± 1.7% α-helices. It was also estimated that the protein has 14.9 ± 4.9% β-turns, and 40.5 ± 6.6% random/unordered secondary structure (Table 2). Likewise, a previous report on the CD spectra of human VDAC (hVDAC1) in DPhPC liposome estimated the β-strand, α-helix, β-turn, and random coil contents of hVDAC1 as 37.3%, 7.7%, 22.6%, and 32.4%, respectively^35^. The association of MBOMP30 in DPhPc liposomes and the far-UV and CD data further support our prediction that MBOMP30 is a β-barrel protein.

### Localization of MBOMP30 to the *Entamoeba* mitosome

**Figure 4:**
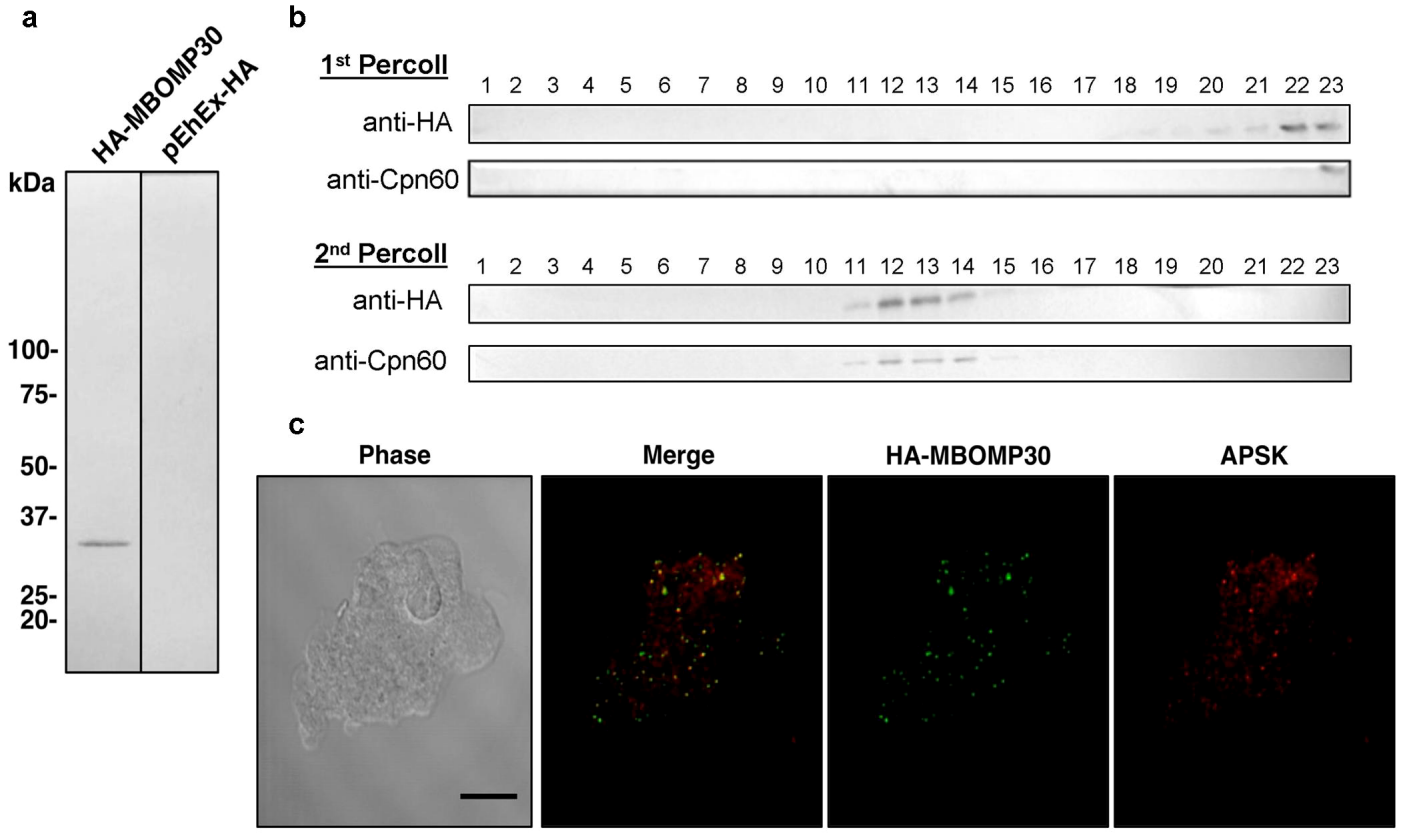
Localization analyses of MBOMP30. (a) Ectopic expression of HA-MBOMP30 in *E. histolytica* trophozoites. Approximately 20 μg of cell lysates from HA-MBOMP30 and control strain was fractionated on SDS-PAGE and subjected to immunoblot analysis using anti-HA antibody. (b) Immunoblot analysis of the fractions by two series of Percoll gradient ultracentrifugation. Approximately 20 μL of each fraction of the first and second ultracentrifugation was separated by SDS-PAGE and blotted to nitrocellulose membranes. The blots were cut into strips containing the region of the target proteins, before reacting with anti-HA and anti-Cpn60 antibodies. (c) Immunofluorescence analysis of HA-MBOMP30 trophozoites. Colocalization of punctate anti-HA (green) and anti-APSK (red; mitosomal marker) signals is shown [scale bar, 10 μm].

We experimentally verified the localization of MBOMP30 in *E. histolytica* trophozoites using an *E. histolytica* cell line expressing MBOMP30 tagged with the hemagglutinin epitope (HA) at the amino terminus (HA-MBOMP30). We avoided potential interference with the putative β-signal, which is located at the carboxyl terminus, by using amino-terminal HA tagging. We confirmed the molecular mass of the expressed product was as expected using whole amoebic lysates and anti-HA antibody (Fig. 4a). We further fractionated lysates from HA-MBOMP30-expressing trophozoites by two rounds of Percoll gradient ultracentrifugation, followed by immunodecoration with anti-HA antibody and antiserum raised against Chaperonin 60 (Cpn60), a canonical mitosomal protein. The distribution of the band corresponding to HA-MBOMP30 throughout the fractions was similar to that of Cpn60 (Fig. 4b), suggesting that MBOMP30 localizes to mitosomes. In addition, immunofluorescence assay (IFA) using anti-HA antibody, and anti-adenosine-5′-phosphokinase (APSK; XP_656278; also mitosomal^18^) antisera showed that HA-MBOMP30 colocalizes with APSK (Fig. 4c). This result confirms the mitosomal localization of MBOMP30 reported previously^18^.

### MBOMP30 is integrated to the mitosomal membrane

**Figure 5:**
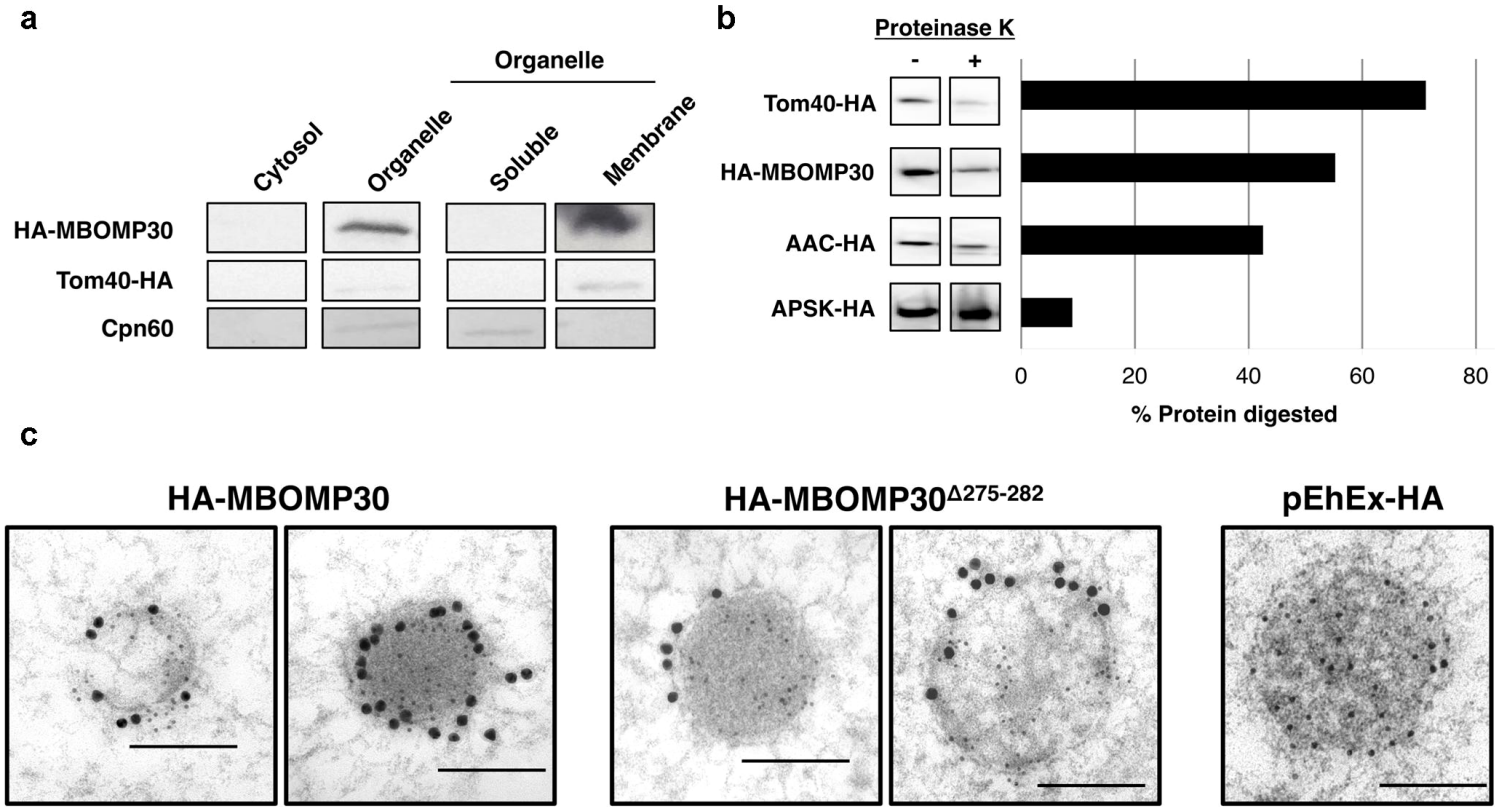
Mitosomal membrane localization of MBOMP30. (a) Na_2_CO_3_ fractionation. Homogenates from amoebae expressing HA-MBOMP30 and Tom40-HA were fractionated. The organelle fraction was treated with Na_2_CO_3_ and NaCl to lyse the organelle and liberate loosely bound proteins. The resulting fractions were separated on SDS-PAGE followed by immunoblotting. Parts of the full-length immunoblot for the anti-HA (first two rows) and anti-Cpn60 (last row) antibody reactions are shown respectively. Tom40-HA and Cpn60 serve as control for mitosomal BOMP and soluble mitosomal protein, respectively. The original blots are shown in Supplementary Fig. S4a–b. (b) Proteinase K protection assay. Cropped immunoblots of proteinase K-treated (+) and untreated (−) organelle fractions are shown on the left panel, with the corresponding ratio of protein digestion on the right panel (See full-length immunoblots on Supplementary Fig. S4c) (c) Immunoelectron microscopy. Immunodecoration of mitosomes of HA-MBOMP30, HA-MBOMP30^Δ275–282^, and control trophozoites with anti-HA (15 nm gold particles) and anti-Cpn60 antibodies (5 nm gold particles) showed electron-dense organelles surrounded by double membranes [scale bar, 200 nm].

To verify our prediction that MBOMP30 is an integral outer membrane protein, we subjected the particulate (membrane) fraction to 100,000 *g* ultracentrifugation with Na_2_CO_3_, which is known to liberate soluble matrix and peripheral membrane proteins from organelles^36^. Immunoblot analysis showed that HA-MBOMP30 was detected in the pellet fraction after Na_2_CO_3_ treatment, similar to the positive control, mitosomal BOMP EhTom40-HA, suggesting membrane integration of HA-MBOMP30 (Fig. 5a). In contrast, the soluble mitosomal matrix protein Cpn60 was observed only in the supernatant fraction after Na_2_CO_3_ treatment (Fig. 5a). We also performed proteinase K protection assay to ascertain the membrane topology of MBOMP30 in amoeba trophozoites. Immunoblots revealed that the sensitivity pattern of MBOMP30 to proteinase K degradation is intermediate to that of an outer membrane control, Tom40-HA, and an inner membrane control, AAC (ATP/ADP Carrier^25^,^37^)-HA, while expectedly, a matrix marker APSK-HA, showed the least susceptibility to proteinase K digestion (Fig. 5b, Supplementary Fig. S3). Interestingly, when we observed mitosomes of HA-MBOMP30 trophozoites by immunoelectron microscopy, it appears that the protein is localized on the membrane of the mitosomes (Fig. 5c). The micrographs reveal mitosomes having an electron-dense region, with a diameter ranging from 100–600 nm, enclosed by a double membrane, with the matrix marked by anti-Cpn60 staining (5 nm gold particles). Notably, abundant anti-HA staining (15 nm gold particles) of mitosomal membranes was observed in HA-MBOMP30-expressing transformants. In addition, the micrographs of randomly selected mitosomes (n = 11) in HA-MBOMP30 trophozoites, revealed that the gold anti-HA particle distribution of 25.2 ± 15.7 gold/μm^2^ in the mitosomal membranes was significantly higher compared to just 0.18 ± 0.22 gold/μm^2^ in the cytoplasm (p < 0.001, using Student's *t*-test).

### MBOMP30 has a putative β-signal

A short, carboxyl-terminal sequence known as the β-signal^38^ plays an important role in MBOMP integration and/or assembly in the mitochondrial outer membrane of *Saccharomyces cerevisiae* and by comparative sequence analysis is inferred to do so in a wide range of eukaryotes. Based on a comparison of numerous putative MBOMP sequences, we have proposed a slightly refined β-signal consensus sequence: P_o_xGh_y_xH_y_xH_y_ (P_o_: non-negatively charged polar residue, G: glycine, H_y_: large hydrophobic residue, h_y_: loosely defined hydrophobic residue including alanine and cysteine, x: any residue, see Materials and Methods)^27^. We also note that the β-signal almost never contains the secondary structure breaker proline in any position. As shown in Fig. 2, *E. histolytica* MBOMP30 has an appropriately placed match to the β-signal, but in *E. invadens* MBOMP30 the match is not perfect, because an alanine is aligned where a polar residue should occur. However, a few MBOMPs with imperfect matches to the β-signal are known, for example the *Saccharomyces pombe* Mdm10 β-signal (FFGVHFEY) has a phenylalanine residue in place of a polar residue in the first position^26^.

Our immunoelectron microscopy results for HA-MBOMP30^Δ275–282^ (HA-MBOMP30 lacking the β-signal) show its localization on the mitosomal membranes (Fig. 5c). Moreover, the gold anti-HA particle distribution in 13 randomly selected micrographs of HA-MBOMP30^Δ275–282^ mitosomes is higher in the mitosomal membranes 13.4 ± 11.9 gold/μm^2^ compared to 0.03 ± 0.07 gold/μm^2^ in the cytoplasm (p = 0.0016, using Student's *t*-test). This data clearly suggests that membrane integration was unaffected even with the truncation of the putative β-signal, suggesting that this sequence is not required for outer membrane targeting per se. This observation is supported by the immunoblot of HA-MBOMP30^Δ275–282^ cells fractions, showing that the protein was detected in the organelle fraction and was retained in the organellar membrane fraction even after treatment with Na_2_CO_3_ (Supplementary Fig. S4a).

### MBOMP30 forms a ~240 kDa protein complex

**Figure 6:**
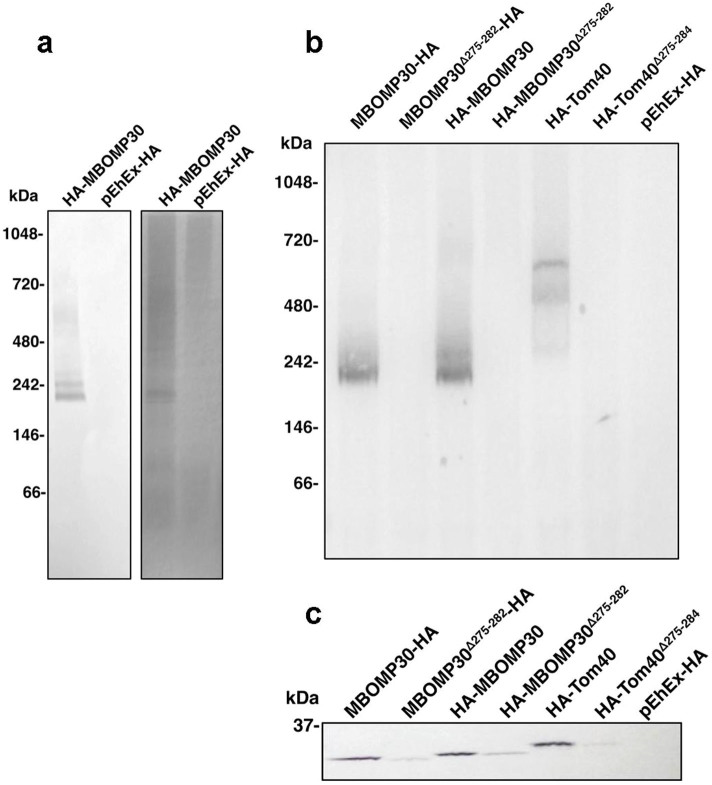
Formation of a 240-kDa EhMBOMP30 complex. (a) Immunoprecipitation of organelle fraction solubilized in 2% digitonin, followed by BN-PAGE and anti-HA immunostaining (left panel) and silver staining (right panel). (b) Anti-HA immunoblots of BN-PAGE and (c) SDS-PAGE of subsequent immunoprecipitations using solubilized organelle fractions from amoeba overexpressing MBOMP30 with HA-tagging at either ends of the protein, with or without the putative β-signal. HA-Tom40 was used as a control.

We investigated whether MBOMP30 forms a complex, by immunoprecipitation of digitonin-solubilized organelle fraction of HA-MBOMP30. By performing blue native polyacrylamide gel electrophoresis (BN-PAGE) of immunoprecipitated samples, followed by immunoblot analysis using anti-HA antibody, we detected a band suggestive of an MBOMP30-containing complex of approximately 240 kDa (Fig. 6a). We also tested if the complex is formed without the putative β-signal sequence. Interestingly, the ~240 kDa complex was not observed in either amino- or carboxy-terminal HA-tagged MBOMP30^Δ275–282^ trophozoites. Furthermore, we also observed a similar phenomenon with immunoprecipitated HA-Tom40 and HA-Tom40^Δ275–284^ from solubilized organelle fractions (Fig. 6b–c). This suggests that the β-signal may have a role in establishing stable MBOMP complex formation in *E. histolytica*.

## Discussion

By a combination of *in silico* and experimental work we have identified a novel eukaryotic subclass of mitochondrion-related-organelle β-barrel protein, found exclusively in the genus *Entamoeba*. MBOMP30 is the seventh MBOMP subclass, lacking any recognizable sequence homology to any of the six previously identified MBOMP subclasses. Although we have not determined the structure of MBOMP30 (e.g. via protein NMR or X-ray crystallography) and therefore our conclusion may not be considered as proven definitively, we have presented a considerable amount of experimental evidence to support our case. First, the *in silico* prediction of the secondary structure ratios by PSI-PRED (Table 1), was strongly corroborated by the deconvolution of the CD and far-UV spectroscopic data of MBOMP30 in DPhPc (Table 2), revealing a high ratio of β-sheet, consistent with the protein being a β-barrel. In a previous report, the CD spectral deconvolution analysis of human MBOMP hVDAC1 in DPhPC was also found to have high β-strand content^35^, similar to what we observed in MBOMP30 and GFP. Second, Percoll gradient ultracentrifugation and Na_2_CO_3_ fractionation experiments showed MBOMP30 behaved like the representative *Entamoeba* MBOMP Tom40^39^. Third, imaging data, provided by immunofluorescence and immunoelectron microscopy, strongly indicate mitosomal, as well as mitosomal membrane localization of MBOMP30. Fourth, the membrane integration of MBOMP30 does not appear to be mediated by lipid modifications. It lacks a canonical amino-terminal secretory signal peptide, thus it is unlikely to go through the ER. Potential lipid modification sites including GPI attachment or isoprenylation are also absent. Highly reliable and well-benchmarked predictors of α-helical transmembrane regions such as Phobius^40^ also predicted no such region in MBOMP30. Moreover, the synthesis of MBOMP30 by an *in vitro* wheat germ translation system demonstrated that it is spontaneously integrated into lipid bilayers (Supplementary Fig. S2), which was similarly observed in other integral mitochondrial membrane proteins such as the VDAC1 in *Homo sapiens*^35^ and the dicarboxylate–tricarboxylate carrier in *Arabidopsis thaliana* and *Plasmodium falciparum*^41^.

The mitosome of *Entamoeba* lacks most of the canonical processes and components existing in the mitochondrion and even the hydrogenosome^3^. In particular, the mitosomal outer and inner membranes appear almost bare, lacking homologs for most of the components associated with protein import. The assembly of BOMPs typically requires the outer membrane complexes TOM and SAM, containing the β-barrel proteins Tom40 and Sam50 respectively^6^. Recently, Tom60^39^, a component unique to the *Entamoeba* TOM complex, was discovered. This essential tetratricopeptide repeat-containing protein acts as both a cytoplasmic carrier of soluble and membrane premitosomal proteins, as well as a lone structural component of the *Entamoeba* TOM complex. Aside from Tom60, the *Entamoeba* genome contains no detectable homologs to the non-β-barrel proteins which usually work in concert with Tom40 and Sam50^3^,^29^, including the small Translocase of the Inner Membrane (small TIM) complexes Tim9-Tim10 and Tim8-Tim13 required for translocation of precursor BOMPs from the TOM to the SAM complex^6^,^7^. Also, there appears to be no homolog of Sam35 in *E. histolytica*, the non-BOMP subunit of the SAM complex which recognizes the β-signal in yeast prior to BOMP assembly via Sam50^38^.

Interestingly, the *E. histolytica* MBOMP30 and Tom40 (Supplementary Fig. S5) match our slightly refined β-signal consensus sequence. However, the *E. histolytica* Sam50 homolog does not have anything close to a β-signal match, but instead has a terminal phenylalanine, which (although possibly coincidental) matches the membrane integration/assembly signal for bacterial BOMPs, simply consisting of a large aromatic residue [FYW] as the final residue of the protein^42^ (Supplementary Fig. S5). This observation is intriguing in its own right, as Sam50 is an MBOMP with a recognizable bacterial BOMP homolog (Omp85/BamA), which itself typically ends in [FYW]. Given that the *Entamoeba* proteome seems to exhibit an unusual mixture of systems of diverse phylogenetic origin^18^, it is conceivable that their MBOMPs utilize both eukaryotic and bacterial mechanisms for membrane insertion. The existence of MBOMPs that do not possess the β-signal motif like *E. histolytica* Sam50, suggests that there is a possibility of finding further candidates that also do not match the β-signal motif. Thus, we also performed an *in silico* screening using our MBOMP predictor without the β-signal related features. Unfortunately, this screening yielded only the known MBOMP EhSam50 and EHI_062770, a protein with a C2 domain forming a beta-sandwich fold.

To investigate the role of the putative *Entamoeba* β-signal in BOMP assembly we transfected amoebic trophozoites to overexpress MBOMP30 lacking the β-signal. Interestingly, our data from sodium carbonate fractionation and immunoelectron microscopy of HA-MBOMP30^Δ275–282^ both indicate integration of the protein to the mitosomal membrane. From a survey of the immunoelectron micrographs of several randomly selected mitosomes, HA-MBOMP30 was observed to have double the number of anti-HA gold particles compared to the β-signal-truncated overexpressor. The decrease in the number of detected HA-particles may be due to a generally lower expression level of the protein lacking the β-signal, or the cytosolic degradation of the translated mutant protein after unsuccessful integration to the mitosomal membrane. Nevertheless, the protein is highly concentrated on the mitosomal membrane compared to the cytoplasm, regardless of the presence or absence of the β-signal, suggesting that this sequence may not be exclusively essential for proper integration of the protein to the mitosomal membrane but may serve other potential roles.

Incidentally, data from our immunoprecipitation experiments using showed that the deletion of the β-signal in MBOMP30 disrupted the formation of the ~240 kDa complex. Similarly, abolishment of the formation of a 600 kDa TOM complex was observed in *Entamoeba* Tom40 lacking the β-signal (Fig. 6b), suggesting that the β-signal may be required for sorting, formation, and/or stability of complex formation in *Entamoeba* MBOMPs. Although an exact role of the β-signal in the MBOMP30 complex formation remains unknown, different mechanisms for recognition, sorting, and integration of β-barrel proteins possibly exist in the mitosomes of *Entamoeba*. It is plausible that MBOMP30 release from either Tom40 or Sam50 is blocked by the deletion of its β-signal, preventing it to properly form the ~240 kDa complex on the outer membrane. A similar phenomenon was observed when truncation of some residues of the β-signal in yeast Tom40, Mdm10, and porin, did not impair binding to Sam50, but prevented formation of the TOM complex on the outer membrane^38^. It is tempting to speculate that, with the minimal components for protein import, especially BOMP assembly, the *Entamoeba* mitosome has developed simplistic alternative mechanisms of membrane protein translocation and integration, and/or that possible functional homologs of “missing” components associated with protein import, are yet to be discovered.

*Entamoeba* mitosomes compartmentalize enzymes required for sulfate activation. Since *Entamoeba* mitosomes transport substrates and produce intermediary metabolites of sulfate activation, such as sulfate, adenosine phosphates, phosphate, and 3′-phosphoadenosine-5′-phosphosulfate (PAPS), it is expected that they possess suitable transporters in their membranes. However, a sensitive hidden Markov model-based similarity search failed to find an *Entamoeba* homolog of VDAC, the usual channel for metabolites in the outer membrane of mitochondria^43^. Without VDAC, it is unclear how metabolites are transported across the outer membrane in this organism. This study is partially motivated by our speculation that novel transporters may serve this role, although it is also possible that Tom40 serves as the transporter of both proteins and metabolites, as it is known to transport some small molecules in yeast^44^,^45^. In the same manner, there is also a possibility that MBOMP30 has function similar to those of the MBOMPs, Mdm10 or Tac40. In yeasts, Mdm10 tethers the mitochondria to the ER and is involved in the ERMES^12^. Several cellular processes are associated with this BOMP, including the maintenance of mitochondrial morphology, and Ca^2+^ transport and lipid exchange between the ER and mitochondria. Although there are no detectable *Entamoeba* homologs of the proteins comprising ERMES^46^, such as the Maintenance of Mitochondrial Morphology protein 1 (Mmm1), Mdm12, and Mdm 34^12^, it is conceivable that MBOMP30, like Mdm10, may be involved in linking the mitosome to the ER via unknown functional homologs, to other *Entamoeba* organelles, or even to cytoskeletal proteins, as Mdm10 binds to actin^47^ and Tac40 attaches to the basal body of the *T. brucei* flagellum^14^.

Our repeated attempts at knocking down expression of MBOMP30 by gene silencing have failed, suggesting that the protein is central and essential to the survival of *E. histolytica* trophozoites, and in good contrast to the fact that the knockdown of chaperonin 60, ADP/ATP carrier, and three enzymes involved in sulfate activation was not lethal^25^. We have also attempted to identify the components of the ~240 kDa complex. Several ER and cytoplasmic proteins were detected in the MS-MS analysis. Converse immunoprecipitation yielded a complex with almost identical size, however MS-MS sequencing did not detect the presence of MBOMP30. There is a possibility that the protein exists as a homo-oligomer, like bacterial BOMPs such as MspA, a porin uniquely found in *Mycobacterium smegmatis* that forms a homo-octameric complex^48^.

In conclusion, the discovery of MBOMP30 represents only the seventh class of eukaryotic MBOMPs and therefore significantly increases our understanding of the range of sequence and phylogenetic distribution possible for this structural class of eukaryotic protein. Although BOMPs are numerous and diverse in bacteria^49^, systematic attempts to find them in eukaryotic genomes have not yielded novel MBOMPs^26^,^27^,^50^ so far. However, it is important to remember that the diversity of eukaryotes, and consequently the diversity of mitochondria and MROs, remains hidden in organisms whose genomes are yet to be completely sequenced. This study could potentially aid and stimulate further searches for novel MBOMPs. In addition to shedding light on MBOMPs in general, discovery of the lineage-specific MBOMP30 will help guide experiments to elucidate its function and better understand the biology and evolution of the Amoebozoa.

## Methods

### MBOMP prediction pipeline

For MBOMP prediction we used a modified version of our Support Vector Machine (SVM)-based predictor developed for a previous study^27^. Like other BOMP predictors^51^,^52^, our predictor considers physicochemical features reflecting BOMP structural motifs (such as β-strands with alternating hydrophobic residues) expected to be found in the β-barrel region. Unique to our predictor is the consideration of sorting signals; e.g. the β-signal, which MBOMPs might be expected to have, and signal peptides which MBOMPs are expected *not* to have (Fig. 1). For this study we trained our predictor on a dataset including mitosomal and hydrogenosomal BOMP sequences, to improve its ability to detect novel BOMPs in MROs. Although not packaged for easy use, the source code for our predictor is available upon request.

The first step in our pipeline requires a relatively high MBOMP probability score (probability > 0.7) from our MBOMP classifier. We additionally require the secondary structure composition as predicted by PSI-PRED^53^ to be at least 25% β-strand and no more than 25% α-helix.

#### Training dataset

We prepared a dataset of 81 MBOMP sequences (including presumed MBOMPs inferred by sequence similarity), consisting of 71 mitochondrial BOMPs and 10 MRO-BOMPs: Tom40 and Sam50 from *E. histolytica, E. invadens, Giardia intestinalis, Encephalitozoon cuniculi, Cryptosporidium parvum, Trichomonas vaginalis* and *Blastocystis hominis*. No pair of positive examples shared more than 40% identity. For negative examples we used 2464 non-MBOMP yeast proteins with clear Uniprot annotation and less than 20% mutual sequence identity. The sequence data were obtained from Uniprot and EuPathDB^54^,^55^. This dataset is available upon request.

#### Newly integrated β-signal and physicochemical sequence features

As shown in Supplementary Fig. S5, we observed that most MRO-BOMPs in our training set contained matches to the β-signal, therefore we decided to add β-signal-inspired features to our MBOMP predictor via two representations: Position Weight Matrix (PWM) and regular expression. To define our PWM based β-signal inspired features, we first divided our 81 training MBOMPs by protein family and used MAFFT^56^ to obtain four multiple alignments (one for each MBOMP family). We then extracted a total of 81 aligned regions (octomers) matching the β-signal from the multiple alignments and discarded duplicate octomers to obtain 71 β-signal examples. We then defined a PWM based on those 71 examples and background frequencies as described in our previous study^27^. Finally, we defined two features derived from this PWM; its maximum value over the C-terminal 50 residues and over the entire sequence.

We also defined five binary features based on the occurrence of matches in the C-terminal 50 residues to regular expressions representing the β-signal motif. For these regular expressions we used the original pattern P_o_xGxxH_y_xH_y_, as proposed by Kutik et al.^38^ and four variants: P_o_H_y_Gh_y_xH_y_xH_y_, P_o_H_y_Gh_y_Ĥ_y_H_y_xH_y_, Ĥ_y_H_y_Gh_y_xH_y_xH_y_ and Ĥ_y_H_y_Gh_y_Ĥ_y_H_y_xH_y_, proposed in our previous work^27^. In these expressions x is any residue, H_y_, h_y_ and P_o_ denote [VLIMFYW], [ACVLIMFYW], and [KRHSTNQ], respectively, and Ĥ_y_ matches any residue not included in H_y_.

To integrate physicochemical sequence features, we employed an easily implemented method, potentially enabling the SVM to automatically learn the characteristics of various sorting signals based on the physicochemical properties of sequence segments. We partitioned the amino- and carboxyl-terminal 90 residues into 6 blocks of 15 residues, and then computed the average hydrophobicity, α-helical periodicity score^57^, and the density of positive, negative, and aromatic residues for each block. These features are also relevant to structural motifs.

### MBOMP30 homolog search, sequence comparison and topology prediction

We searched for MBOMP30 homologs in other organisms using the JackHMMR and HHblits algorithms^31^,^58^. We computed pairwise alignments and E-values of MBOMP30 homologs among *E. histolytica*, *E. dispar* and *E. invadens* with the SSEARCH program^58^, and built multiple alignments with Clustal omega^59^. For topology prediction of MBOMP30 and its homologs, we used BOCTOPUS and TMBETAPRED-RBF^33^.

### Cell-free synthesis of EhMBOMP30 proteoliposomes

The open reading frame of the *E. histolytica* MBOMP30 gene EHI_178630 was codon-optimized for wheat-germ expression, and cloned to pY08 vector using *Spe*I and *Sal*I restriction sites. Messenger RNA was synthesized by *in vitro* transcription as previously described^60^. EhMBOMP30 was synthesized by wheat-germ expression system^60^, in the presence of 0.5 mg/mL DPhPc liposomes (Avanti Polar Lipids, Alabaster, AL).

### Far-UV and circular dichroism spectroscopy analysis of MBOMP30 proteoliposomes

Synthesized *E. histolytica* MBOMP30 proteoliposomes were pelleted, then washed twice with CD buffer (25 mM sodium phosphate buffer, pH 7.0) by centrifugation at 15,000 rpm at 4°C for 20 min. The pellet was resuspended in 150 μL CD buffer and sonicated briefly until translucent. Recombinant His-tagged GFP was used as a positive control. CD measurements were performed using a Jasco J-820 Spectropolarimeter, and the samples were loaded onto a quartz cuvette with 0.1-cm path length (Starna Cells, Inc, Atascadero, CA). The far-UV spectra (185 to 250 nm) were obtained by taking the average of 9 consecutive scans with a resolution of 1 nm, a scanning speed of 200 nm/min and a response time of 2 s. The secondary structure was predicted by the CONTIN algorithm^61^,^62^ using a 42-protein set database available through the Dichroweb online tool^63^,^64^.

### Plasmid construction

Total RNA was isolated from *E. histolytica* trophozoites by TRIZOL® reagent (Invitrogen, Carlsbad, San Diego, CA). mRNA was purified using GenElute™ mRNA Miniprep Kits (Sigma-Aldrich, Japan). cDNA was synthesized from mRNA using SuperScript™ III RNase H^−^ reverse transcriptase (Invitrogen), and oligo(dT)_20_ primer (Invitrogen). The *E. histolytica* gene EHI_178630 was PCR-amplified from cDNA using Phusion DNA polymerase (New England Biolabs, Beverly, MA) using the following primer sets (sense primer; 5′- GTTCCCGGGATGTTGGGTAAAACTGC -3′ and antisense primers; 5′- GAACTCGAGTTAAAGTGATAAATCAATTCCA -3′ or 5′- GAACTCGAGTTAGAGTTGATTTTTCTGGTCTT -3′) for full length (HA-MBOMP30) or β-signal-truncated (HA-MBOMP30^Δ275–282^) inserts respectively. After restriction digestion, amplified fragments were ligated into pEhEx-HA^65^ using Ligation-Convenience Kit (Nippongene, Tokyo, Japan).

### Cell culture and amoeba transformation

Trophozoites of *Entamoeba histolytica* HM-1:IMSS cl6^66^ and G3^67^ strains were cultivated axenically in Diamond BI-S-33 medium^66^. Lipofection of amoebic trophozoites, selection, and maintenance of transformants were performed as previously described^18^.

### Preparation of organelle fraction

Amoeba strains that expressed HA-MBOMP30, Tom40-HA^39^, AAC-HA^18^, and APSK-HA^18^ proteins, and also the mock transformant, pEhEx-HA, were washed three times with 2% glucose/PBS. After resuspension in lysis buffer (10 mM MOPS-KOH, pH7.2, 250 mM sucrose, protease inhibitors), cells were disrupted mechanically by a Dounce homogenizer. Unbroken cells were removed by centrifugation at 5,000 *g* for 10 min, and the supernatant centrifuged at 100,000 *g* for 60 min to separate the organelle and cytosolic fractions. The 100,000 *g* organelle fractions were resuspended with lysis buffer, and were recollected by centrifugation at 100,000 *g* for 60 min.

### Percoll-gradient and sodium carbonate-mitosomal membrane fractionation and immunoflourescence assay

Percoll-gradient fractionation^18^, Na_2_CO_3_ treatment of mitosomal membrane fractions^39^ was performed as previously described but using anti-Cpn60 as a soluble mitosome protein marker. IFA^18^,^68^ was performed as previously described.

### Proteinase K protection assay

Proteinase K assay was performed following the previous protocol with some modifications^39^. Briefly, organelle fractions (100 μg protein each) were prepared, then treated with proteinase K (the final concentration was 3.125 μg/ml) (Roche) for 15 min at 4°C. Control samples without proteinase K were likewise prepared. Samples were run on SDS-PAGE followed by immunoblot analysis using anti-HA mouse monoclonal antibody and HRP-conjugated anti-mouse antibody (Thermo Scientific) as primary and secondary antibodies respectively. Quantitation of band intensities was performed using the Analysis Toolbox in ImageQuant TL software (GE Healthcare).

### Immunoelectron microscopy

Sample preparation was carried out as previously described^25^ with some modifications. Trophozoites overexpressing HA-MBOMP30 and HA-MBOMP30^Δ275–282^ in BIS medium were incubated with gold disks at 35.5°C for 15 minutes to allow for attachment. The disks carrying amoebae were then frozen in liquid propane at −175°C. Once frozen, the samples were freeze-substituted with 0.2% glutaraldehyde in ethanol and 3% distilled water at −80°C overnight. Sample fixation and sectioning were performed as described previously^25^. The disks were then double-stained using anti-HA mouse and anti-Cpn60 rabbit antibodies in 1% BSA-PBS for 90 min at room temperature, then rinsed with 1% BSA-PBS 3 times for 1 min each. Then, they were reacted with secondary anti-mouse and anti-rabbit IgG antibody conjugated to gold particles for 1 hr at room temperature. They were rinsed and placed in 2% glutaraldehyde in 0.1 M phosphate buffer before drying. Then, the grids were stained with 2% uranyl acetate for 15 min, and secondary-stained with lead stain solution at room temperature for 3 min. The grids were observed at Tokai Microscopy Inc. (Nagoya, Japan), using a transmission electron microscope (JEM-1400 Plus, JEOL Ltd.,) at an acceleration voltage of 80 kV. Digital images (2048 × 2048 pixels) were taken with a CCD camera (VELETA, Olympus Soft Imaging Solution GmbH).

### Immunoprecipitation of the EhMBOMP30 complex

Organelle fractions of HA-MBOMP30 and HA-MBOMP30^Δ275–282^ overexpressing strains, and pEhEx-HA control were prepared. Immunoprecipitation was performed as previously described, followed by BN-PAGE and immunoblot analysis^39^.

## Author Contributions

H.S. did the biochemical and cell biological experiments. K.T. and K.I. performed the *in silico* analyses. P.H. contributed to planning the *in silico* part of this study. H.S., T.M. and T.N. designed the study and analyzed data. H.S., M.I., A.N. and Y.T. performed cell-free expression and CD spectroscopy experiments. H.S., K.T., K.I., P.H., T.M. and T.N. wrote the paper.

## Supplementary Material

Supplementary Information Supplementary Materials

## Figures and Tables

**Table 1:** List of predicted MBOMP candidates in *E. histolytica*. JackHMMER searches were performed against NCBI non-redundant protein database with the best E-value amongst annotated proteins shown. The β-signal motif (P_o_xGxxH_y_xH_y_) was matched against the β-strand closest to the C-terminus, as predicted by BOCTOPUS

Protein ID	Length	MBOMP prediction probability	Predicted β-strand ratio	Predicted α-helix ratio	Annotation inferred from homology	JackHMMER Hit (E-value)	Pfam domain (E-value)	β-signal motif hit
EHI_104420	284	0.961	0.29	0.21	Tom40	*C. parvum*, Tom40p like translocase (0.026)	Eukaryotic porin (2.9e-09)	Yes
EHI_178630	282	0.954	0.34	0.23	Unknown	No significant hit	No significant hit	Yes
EHI_007460	304	0.871	0.27	0.20	Phospholipase C	*T. thermophile*, Endonuclease/Exonuclease/phosphatase family protein (1.2e-76)	Endonuclease/Exonuclease/phosphatase family (3.5e-15)	Yes
EHI_163510	263	0.853	0.31	0.18	Exosome complex exonuclease RRP4	*D. rerio*, Exosome complex exonuclease RRP4 (8.9e-100)	S1 RNA binding domain (0.19)	No
EHI_050690	321	0.749	0.43	0.09	Apyrase	*P. sergenti*, salivary apyrase (3.1e-79)	Apyrase (5.4e-09)	Yes
EHI_068370	466	0.734	0.56	0.00	Cysteine protease binding protein family like protein	*E. histolytica*, cysteine protease binding protein family 4 (8.5e-178)	No significant hit	No

**Table 2:** Estimation of EhMBOMP30 secondary structure organization based on CD spectra analysis using CONTIN software

Trial	Predicted β-strand %	Predicted α-helix %	Predicted β-turns %	Predicted random coil %	NMRSD	λ_min_, nm	λ_max_, nm
1	33.5	12.2	19.2	35.1	0.059	221.5	196.0
2	27.8	14.8	9.5	47.9	0.088	221.9	192.5
3	30.3	15.6	15.9	38.5	0.094	220.9	192.8
Avg ± SD	30.5 ± 2.9	14.1 ± 1.7	14.9 ± 4.9	40.5 ± 6.6			

NMRSD is the normalized root mean square deviation from the CONTIN analysis and should be <0.1.
