# Supplementary material for: A Novel Mitosomal β-Barrel Outer Membrane Protein in Entamoeba
Source: Sci Rep. 2015 Feb 25;5:8545. doi: 10.1038/srep08545 (PMC4339806; doi:10.1038/srep08545)
Supplement: Supplementary Information — Supplementary Materials [file srep08545-s1.pdf]

Supplemental Material for "A Novel Mitosomal β-Barrel Outer Membrane Protein in *Entamoeba*"

Herbert J. Santos, Kenichiro Imai, Takashi Makiuchi, Kentaro Tomii, Paul Horton,

Akira Nozawa, Mohamed Ibrahim, Yuzuru Tozawa, and Tomoyoshi Nozaki

Cross-Validation Performance of our updated MBOMP predictor

To identify novel MBOMP candidates in the *E. histolytica* genome, we customized our MBOMP predictor[1] for MRO's, as well as refining the method in general (see methods in the main text). Ideally we would benchmark our prediction accuracy with no overlap, in terms of protein families, between training and test sets. Unfortunately, the small number of known MBOMP families makes this impractical. As a compromise, we performed cross-validation using mitochondrial and MRO sequences with relatively low sequence identity (see methods), attaining a precision of 0.96 ±0.03, recall of 0.90 ±0.08, and Matthew's Correlation Coefficient (MCC) of 0.93 ±0.05 in 5-fold cross validation. This represents a rough and (possibly highly) optimistic estimate of our methods prediction accuracy on truly novel MBOMPs.

Prediction of MRO-BOMPs from mitochondria BOMP training set

To explore the potential of our predictor to generalize to MRO-BOMPs, we trained it only on the 71 mitochondrial BOMPs as positive data, and then tested whether it could correctly predict the 10 MRO-BOMPs. This test is not ideal because MRO-BOMPs are in fact defined as such due to distant, but detectable, sequence similarity to mitochondrial proteins. However, this test is not completely trivial because the sequences of MRO-BOMPs are diverse and distinct from mitochondrial BOMPs (e.g.

the sequence identity is less than 25% between *S. cerevisiae* and *E. histolytica* Tom40). In this test our predictor correctly predicted eight of ten MRO-BOMPs.

Empirical p-value of the predictor score attained by EhMBOMP30

To test the risk of finding a high scoring false positive which would also happen to be a mitosomal (but not necessarily outer membrane) protein, we performed a sequence scrambling test using 95 known *E. histolytica* mitosomal proteins[2]. We scrambled the amino acids in each protein sequence, performed MBOMP prediction against the 95 scrambled sequences, and recorded the maximum score achieved. Only 5 of the 100 scrambled sequence trials produced any prediction score higher than the score of the actual EhMBOMP30 sequence. This suggests the risk of finding by chance an equally high scoring false positive amongst known *E. histolytica* mitosomal proteins may be around 5%.

Table S1. Results of α-helical transmembrane, signal peptide and MBOMP prediction on yeast known non-BOMP integral mitochondrial outer membrane proteins.

To assess the risk of wrongly concluding that an α-helical type integral outer membrane protein is an MBOMP, we tabulated the results of the α-helical transmembrane segment predictor Phobius[3] and our MBOMP predictor on known α-helical type integral outer membrane proteins from yeast. For this table we retrained our MBOMP predictor, removing any proteins on this list from the training data. All proteins were assigned very low MBOMP probability scores and all but OM14 are predicted to have at least one transmembrane α-helix or a signal peptide.

Gene Name	Amino Acid Length	Number of Predicted Transmembrane α-helical Regions	Signal Peptide Predicted	Predicted MBOMP Probability
TOM20	183	0	Yes	0.0000
TOM22	152	1	No	0.0000
TOM70	617	0	Yes	0.0000
TOM5	50	1	No	0.0000
TOM6	61	1	No	0.0000
TOM7	60	1	No	0.0037
FZO1	855	1	No	0.0000
UGO1	502	2	No	0.0000
SCM4	187	3	No	0.0000
OM45	393	0	Yes	0.0000
OM14	134	0	No	0.0000

Table S2. Results of EhMBOMP30 homolog search. Using SSEARCH, we were able to identify candidate homologs of EhMBOMP30 limited to the genus *Entamoeba*. Furthermore, we show the results of SSEARCH, targeting specific organisms, clearly indicating the lack of homologs in representative eukaryotes having MROs or mitochondria.

Organisms	Organelle type	Best Hit (length)	E-value	Annotation
Entamoeba nuttalli	Mitosome	ENU1_140620 (283)	3.7e-99	Uncharacterized protein
Entamoeba dispar	Mitosome	EDI_035580 (239)	1.2e-66	Uncharacterized protein
Entamoeba invadens	Mitosome	EIN_041060/ EIN_066350 (286)	1.8e-27	Uncharacterized protein
Giardia lamblia	Mitosome	GSB_150221 (418)	0.46	Uncharacterized protein
Encephalitozoon cuniculi	Mitosome	ECU01_0380 (310)	0.3	Uncharacterized protein
Cryptosporidium parvum	Mitosome	cgd6_5020 (216)	1.4	Protein with WD40 repeats
Trichomonas vaginalis	Hydrogenosome	TVAG_193490 (552)	2.3	Uncharacterized protein
Blastocystis hominis	Hydrogen -producing mitochondria	GSBLH_T0000613 5001 (329)	0.5	Uncharacterized protein
Dictyostelium discoideum	Mitochondria	DDB_0184270 (187)	1.7	Uncharacterized protein
Trypanosoma brucei	Mitochondria	Tb927.3.5550 (183)	0.54	Small GTP-binding protein, putative
Saccharomyces cerevisiae	Mitochondria	GYP1 (637)	0.96	GTPase-activating protein GYP1

a

```
SEQ            MLGKTAPFDTFNFTKQIFDTRNPSPLTLSVNAFGSKTTFGFRESDDTETTPKFTYNSCPQIISKFGYKQIETSLNVSTNSQQ
BOCTOPUS       --------------------------BBBBBBBBBB----------------------------------------------
TMBETAPRED-RBF -------BBBB-----------------BBBBBBBBBBBBB------------------BBBBBBBBBBBBB----------

SEQ            IKVSSSLPFYSFTFDAILKDKTIQKVNVNGVAQIYKNIFIGINYLLPSNKLSPVFIKQNEETPIDDNNETKTSVKELIHGIE
BOCTOPUS       -BBBBBBB---BBBBBBB---------BBBBBBB---BBBBBBBB-----------------------------BBBBBBB-
TMBETAPRED-RBF -BBBBBBBBBBBBBBBBB-----------BBBBBBBBBBBBBBBBB------------------------------------

SEQ            QIQQNSSGFICYQESPYKKYSLFFDYSACIIGARLLRKWNNIIIASEAYFKNKRFEAQTAISSNIQNVGNCKLIITSKKQAI
BOCTOPUS       -------BBBBBBB-----BBBBBBBBBBBBBBB-------BBBBBBB--------BBBBBBB-------------------
TMBETAPRED-RBF ---------BBBBB-BBBBBBBBBBBBBBBBBB---------BBBBBBBBB-------------------------------

SEQ            AQITHPITNLLKLKIQYFHSYEDQKNQLSFGIDLSL
BOCTOPUS       ----------BBBBBBBB---------BBBBBBBBB
TMBETAPRED-RBF --------BBBBBBBBBBBB-----BBBBBBBBB--
```

b

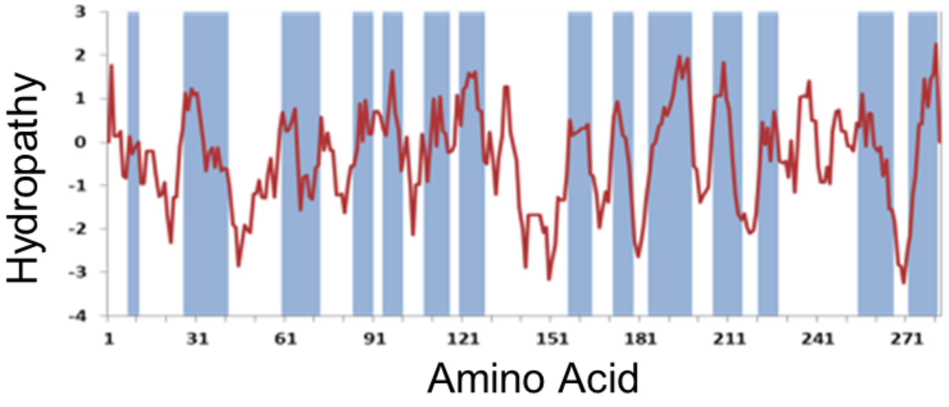

Figure S1. Prediction of transmembrane β-strands and hydrophobicity profile of EhMBOMP30. (a) Residues predicted to be part of a transmembrane β-strand are indicated by a "B" in the track labeled by the prediction method, BOCTOPUS[4] or TMBETAPRED-RBF[5]. (b) Hydropathy profile of EhMBOMP30. The horizontal axis shows the position in the sequence and the vertical axis shows the average hydrophobicity[6] of the 7 residues centered on that position. Blue boxes indicate predicted transmembrane β-strands.

a

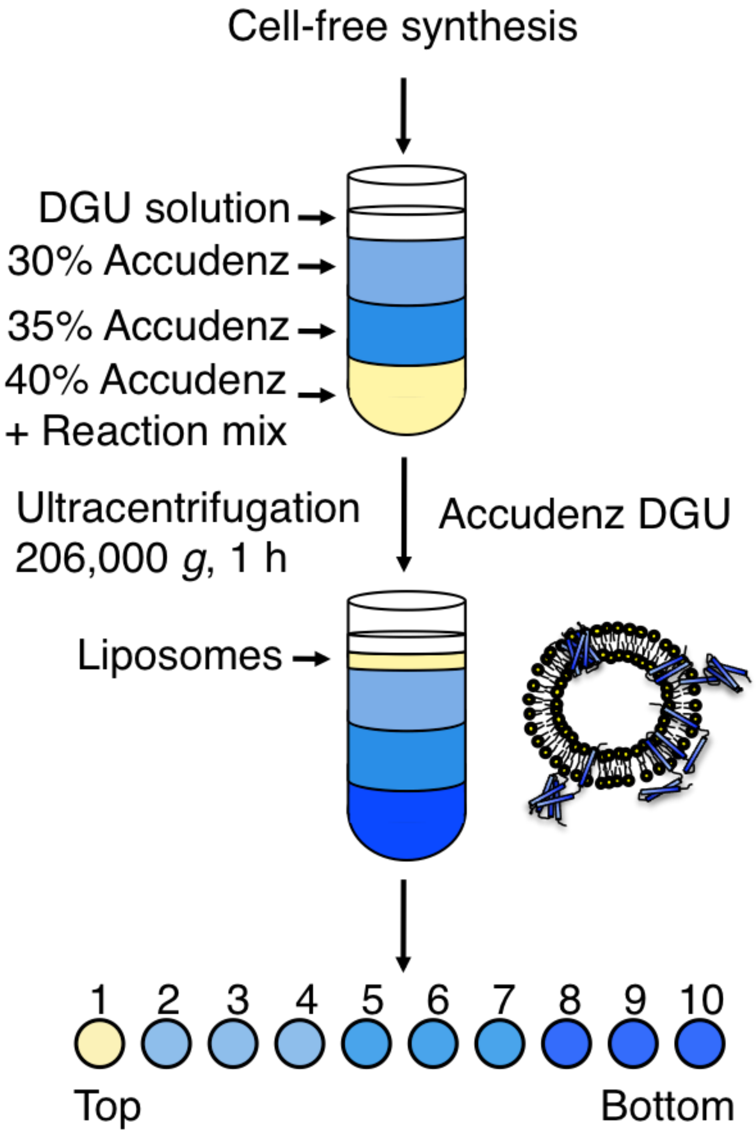
Cell-free synthesis
DGU solution
30% Accudenz
35% Accudenz
40% Accudenz
+ Reaction mix
Ultracentrifugation
206,000 g, 1 h
Accudenz DGU
Liposomes
1 2 3 4 5 6 7 8 9 10
Top
Bottom

b

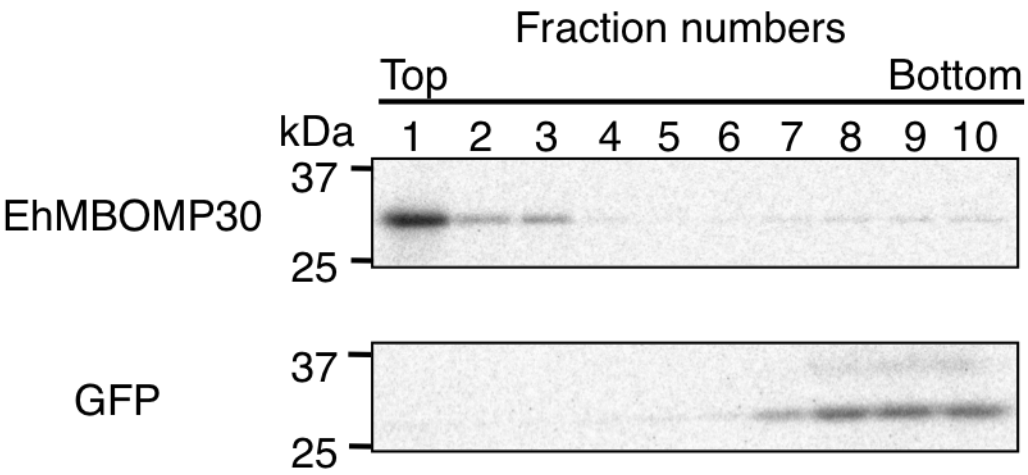
Fraction numbers
Top
Bottom
kDa
1 2 3 4 5 6 7 8 9 10
37
25
EhMBOMP30
37
25
GFP

Figure S2. **Accudenz flotation assay of EhMBOMP30 proteoliposome**. (a) Density gradient ultracentrifugation (DGU) of EhMBOMP30 proteoliposomes, synthesized by cell-free system, in DGU solution and Accudenz (Accurate Chemical and Scientific, Westbury, NY) was performed as previously described[7]. (b) Fractions were collected from the top of the tube and analyzed by SDS–PAGE and autoradiography.

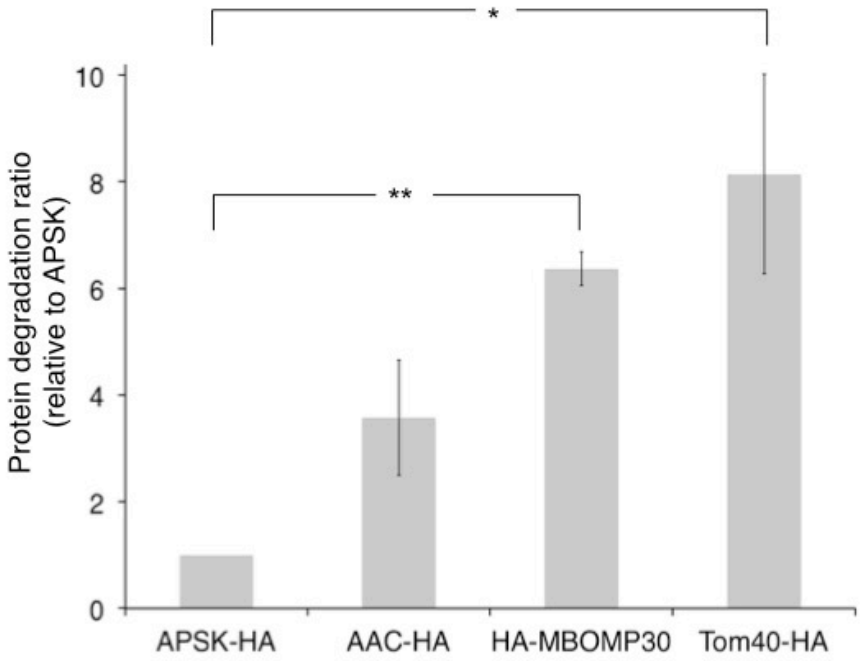

Figure S3. Protease K degradation ratio of HA-MBOMP30 and mitosomal membrane proteins relative to APSK. The percentages of Protease K digestion of HA-MBOMP30, outer membrane control Tom40-HA, and inner membrane control AAC-HA, were normalized against matrix control APSK-HA presented as mean ± standard deviation. The data was analyzed using Student's *t*-test, indicating significant difference between APSK and MBOMP30 (**$p<0.01$) as well as APSK and Tom40 (*$p<0.05$) degradation ratios, strongly suggesting mitosomal membrane and not matrix localization of MBOMP30 based on sensitivity to protease degradation (n=3).

a

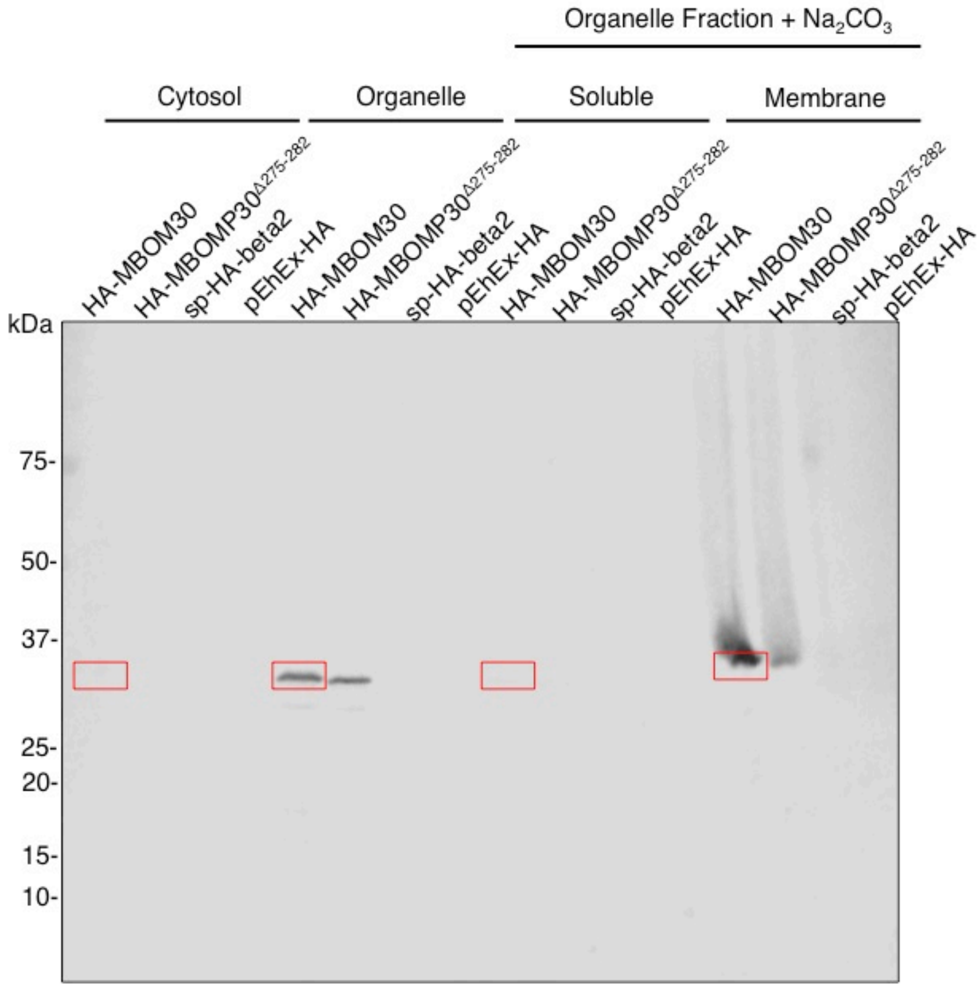

Organelle Fraction + Na_2CO_3
Cytosol
Organelle
Soluble
Membrane
HA-MBOM30
HA-MBOMP30$^{\Delta 275-282}$
sp-HA-beta2
pEhEx-HA
HA-MBOM30
HA-MBOMP30$^{\Delta 275-282}$
sp-HA-beta2
pEhEx-HA
HA-MBOM30
HA-MBOMP30$^{\Delta 275-282}$
sp-HA-beta2
pEhEx-HA
HA-MBOM30
HA-MBOMP30$^{\Delta 275-282}$
sp-HA-beta2
pEhEx-HA
kDa
75-
50-
37-
25-
20-
15-
10-

b

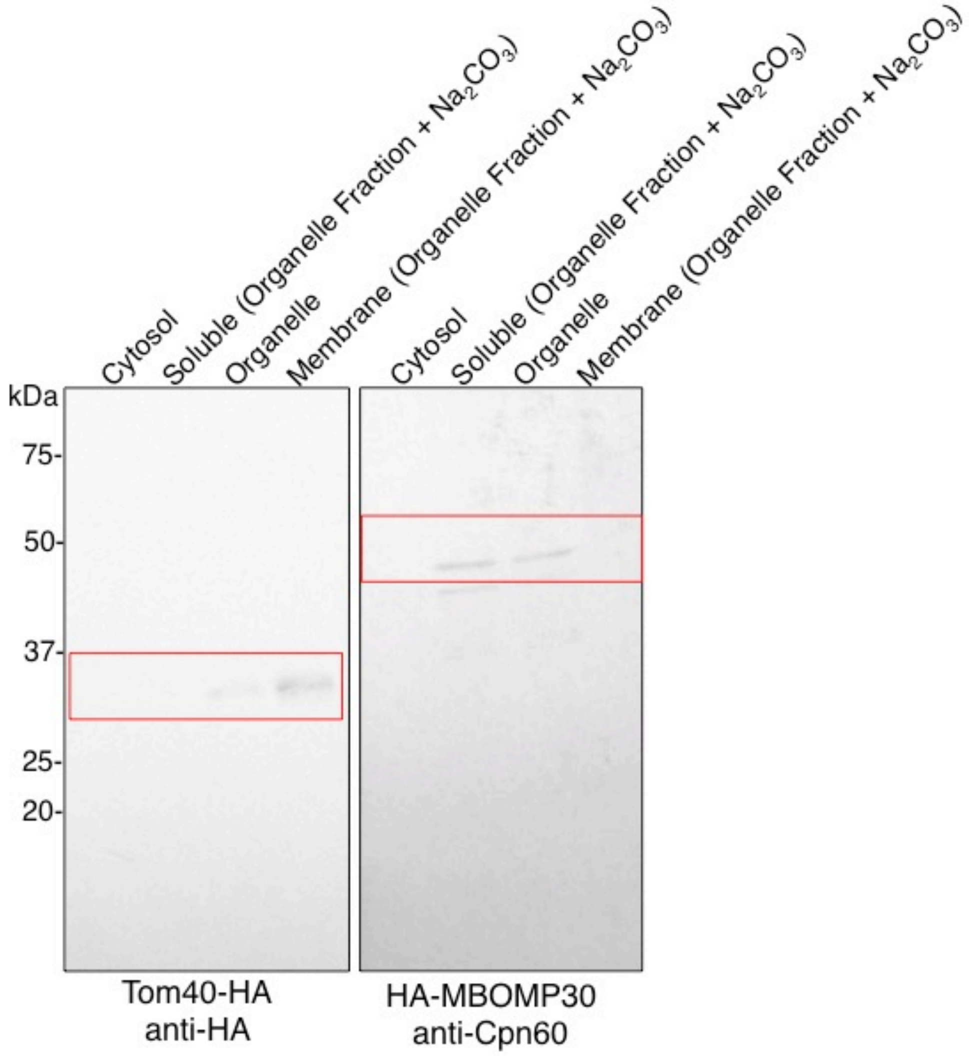

Cytosol
Soluble (Organelle Fraction + Na2CO3)
Organelle
Membrane (Organelle Fraction + Na2CO3)
Cytosol
Soluble (Organelle Fraction + Na2CO3)
Organelle
Membrane (Organelle Fraction + Na2CO3)
kDa
75-
50-
37-
25-
20-
Tom40-HA
anti-HA
HA-MBOMP30
anti-Cpn60

c

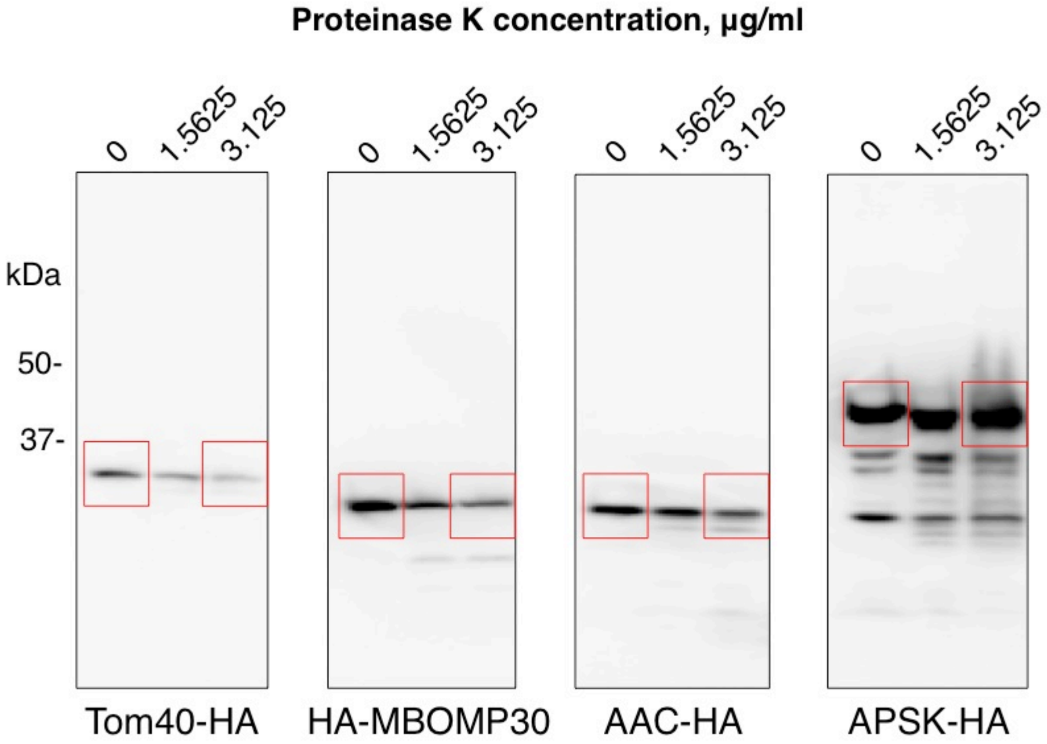

Proteinase K concentration, µg/ml
0
1.5625
3.125
kDa
50-
37-
Tom40-HA
HA-MBOMP30
AAC-HA
APSK-HA

d

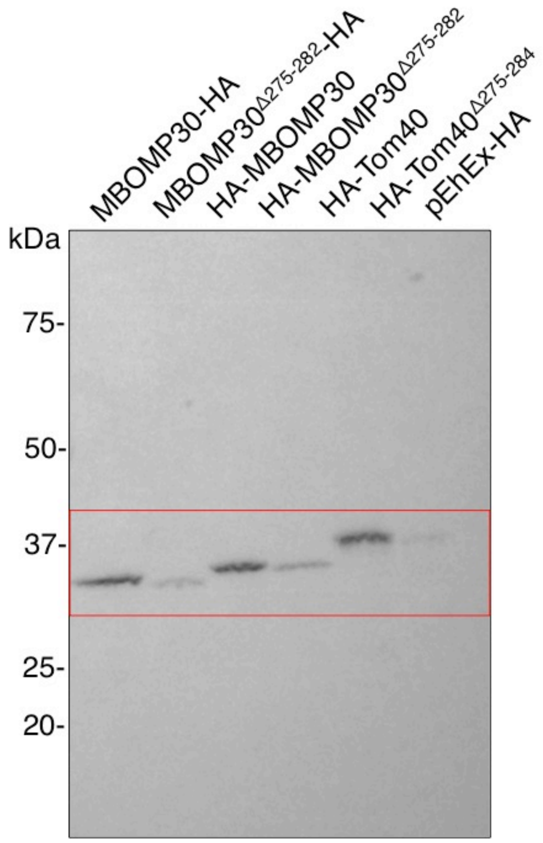

Figure S4. Full-length immunoblots. (a) Results of the anti-HA immunodecoration of sodium carbonate fractionation of HA-MBOMP30. The red boxes indicate the cropped immunoblots shown in the first row of Figure 5a. (b) Sodium carbonate fractionation was performed for Tom40-HA using the same conditions. The immunoblot of Tom40-HA fractions was treated with anti-HA antibody while that of HA-MBOMP30 fractions was reacted with anti-Cpn60. Red crop boxes show the regions presented in the second and third rows of Figure 5a respectively. (c) Organelle fractions of Tom40-HA, HA-MBOMP30, AAC-HA, and APSK-HA were treated with proteinase

K. Cropped boxes show regions of the immunoblots presented in Figure 5b. (d) The SDS-PAGE blot of immunoprecipitated MBOMP30-HA, HA-MBOMP30, and HA-Tom40, with or without the putative β-signal was immunodecorated with anti-HA antibody. The red box highlights the cropped immunoblot shown in Figure 6c.

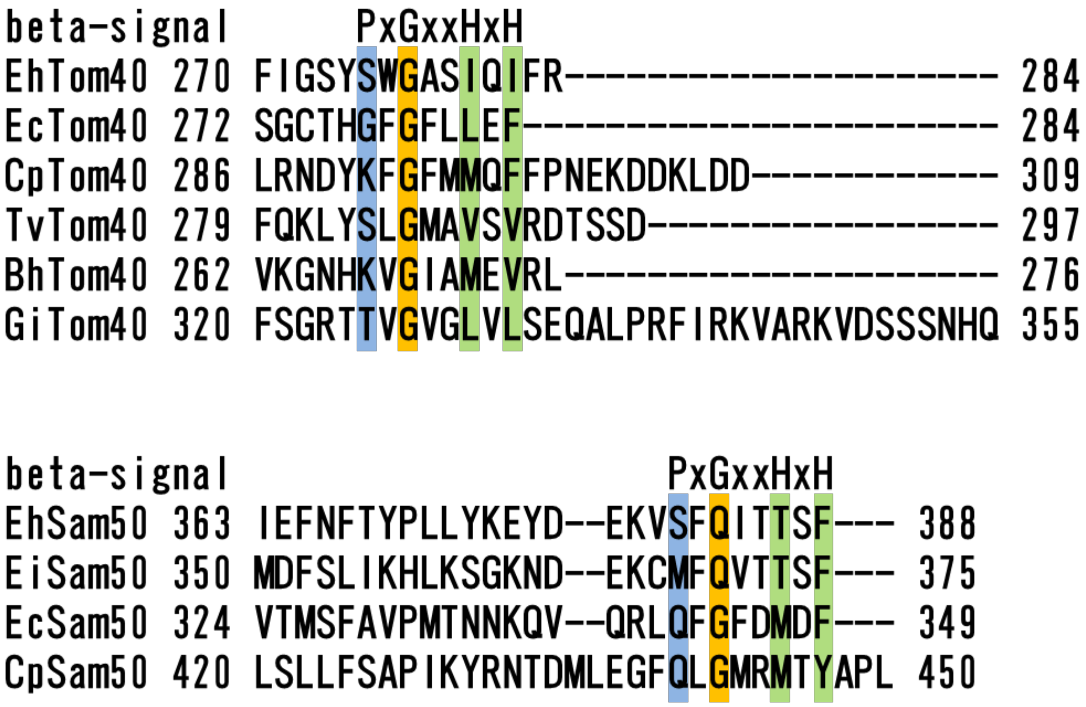

Figure S5. Multiple alignments of potential mitosomal β-signals. Multiple alignments of the vicinity of the β-signal motif matches in MRO orthologs of Tom40 and Sam50 are shown. In EcTom40, EhSam50 and EiSam50, the motif match is not perfect. (Abbreviations: Eh – *Entamoeba histolytica,* Ec – *Encephalitozoon cuniculi*, Ei – *Entamoeba invadens*, Cp – *Cryptosporidium parvum*, Tv – *Trichomonas vaginalis*, Bh – *Blastocystis hominis*, Gi – *Giardia intestinalis*)

References

1 Imai, K., Fujita, N., Gromiha, M. M. & Horton, P. Eukaryote-wide sequence analysis of mitochondrial beta-barrel outer membrane proteins. *BMC Genomics* **12**, 79, doi:10.1186/1471-2164-12-79 (2011).

2 Mi-ichi, F., Abu Yousuf, M., Nakada-Tsukui, K. & Nozaki, T. Mitosomes in Entamoeba histolytica contain a sulfate activation pathway. *Proc Natl Acad Sci USA* **106**, 21731-21736, doi:10.1073/pnas.0907106106 (2009).

3 Kall, L., Krogh, A. & Sonnhammer, E. L. A combined transmembrane topology and signal peptide prediction method. *J Mol Biol* **338**, 1027-1036, doi:10.1016/j.jmb.2004.03.016 (2004).

4 Hayat, S. & Elofsson, A. BOCTOPUS: improved topology prediction of transmembrane beta barrel proteins. *Bioinformatics* **28**, 516-522, doi:10.1093/bioinformatics/btr710 (2012).

5 Ou, Y. Y., Chen, S. A. & Gromiha, M. M. Prediction of membrane spanning segments and topology in beta-barrel membrane proteins at better accuracy. *J Comput Chem* **31**, 217-223, doi:10.1002/jcc.21281 (2010).

6 Kyte, J. & Doolittle, R. F. A simple method for displaying the hydropathic character of a protein. *J Mol Biol* **157**, 105-132 (1982).

7 Nozawa, A., Fujimoto, R., Matsuoka, H., Tsuboi, T. & Tozawa, Y. Cell-free

synthesis, reconstitution, and characterization of a mitochondrial dicarboxylate-tricarboxylate carrier of Plasmodium falciparum. *Biochem Bioph Res Co* **414**, 612-617, doi:10.1016/j.bbrc.2011.09.130 (2011).
